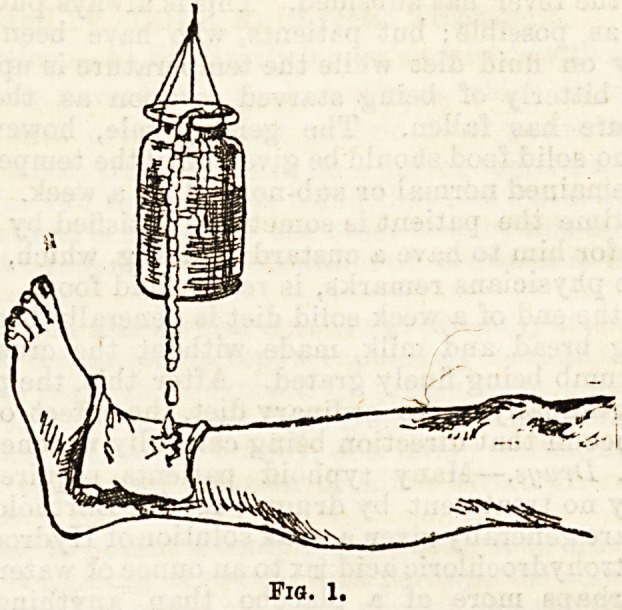# The Treatment of Sprains of the Ankle

**Published:** 1892-10-22

**Authors:** 


### THE QUEEN'S HOSPITAL, BIRMINGHAM.

### The Treatment of Sprains of the Ankle.

As the suitability of the method of treatment to be adopted in each particular case is dependent, to a large extent, on the exact nature of the injury with which we have to deal, it becomes essential to arrive at a little more definite conception of the injury than is conveyed by the term "sprain," which is suggestive neither of the condition of the lesion, nor of the line of treatment to be followed. A sprain has been defined as a temporary dislocation of a joint, and the result of the separation of those articular surfaces normally in close apposition to one another must necessarily be associated with some distinct lesion to those structures which aid directly or indirectly in maintaining such apposition. The chief lesions which thus occur are—(1) Rupture of the ligamentous fibres; (2) rupture of muscular fibres; (3) rupture of the connective tissue structures, such as the fasciæ; (4) displacement of the tendons of the muscles; and these are all more or less associated with rupture of the capillaries leading to extravasation into the soft tissues and intervening spaces. A tolerably accurate localisation of the structures involved may be obtained from pain on applying gentle pressure, indicating the situation of the injured parts, whilst pain is also felt at these spots when passive movements place such structures on the stretch. If it is found that any tendon is displaced this should first be manipulated back into its normal position, and the limb bandaged so as to retain it in position, and at the same time reduce the resulting swelling.

We will now consider the treatment of those sprains which are seen soon after the accident, or in which the amount of effusion is but slight. In these cases, unless the pain is very acute, the application of cooling agents may be altogether dispensed with, as by far the most reliable factors in reducing the existing swelling and preventing its increase are rest, elevation, and pressure. For this purpose the ankle joint should be at once immobilised, and the method adopted is the following: First, place the foot at right angles with the leg, as then undue tension on any of the torn structures is avoided. Then take cotton wool and roll it evenly round the foot and leg to a little above the knee, leav- the toes uncovered; the thickness of the layer of cotton wool should be sufficiently great as to enable pressure being applied evenly, as the success of the treatment depends primarily upon the support which is given to the normal depressions in the neighbourhood of the ankle-joint, and if only a thin layer of wool be used, then only the more prominent parts will receive support, whilst the intervening depressions, being unsupported, will readily permit effusion to occur there. Having, therefore, well encircled the limb with wool, an external and an internal L-shaped splint of millboard should then be applied, and securely fixed by roller bandages. The splints should be long enough to include the knee-joint, especially if the patient is at all restless; the foot-piece should extend to the base of the toes, but not beyond, as it is advisable to leave the toes exposed in order that they may be examined from time to time, lest the amount of extravasation, combined with the pressure of the bandage, should cause obstruction to the circulation. The patient should rest either in bed or on a couch, with the limb slightly elevated on a pillow. If the pain should be at all severe, an ice-bag may be placed over the front of the ankle, and, as a rule, will be sufficient to relieve it. At the end of twenty-four hours the bandages may have become slack, and will require to be reapplied. At the end of forty-eight hours the bandages, splints, and wool should be removed, and the joint examined, as by this time all tendency to effusion will have ceased. Very gentle passive movement may be made, but should not be carried past the limit indicated by pain, and it should only be employed for a short time, after which linimentum iodi should be painted over the affected part, and then the wool, &c., reapplied. Passive movement should be repeated on the fourth day, and again on the sixth day, when the splints may be dispensed with. On the eighth day the patient ought to be able to bear the weight of the body upon that limb, and from that date he should be encouraged to perform active movements.

In those cases in which the amount of extravasation is already very marked or is rapidly increasing, and where the pain is also very severe, it will be found advisable to adopt certain measures as a preliminary to that of bandaging, and for such cases it is usual to apply cold to the part in some form or other, and the following methods are those chiefly in use.

I. Leave leg exposed, but steadied if necessary by sandbags; over the ankle is placed a piece of lint, which is kept continually wet with some evaporating lotion, and for this purpose one of the following formulæ may be employed: (*a*) R. Sp. vini rectific., ℥j.; liq. ammon. acet., ℥i; aq. camphoræ ad ℥viij. (*b*) R. Liq. plumb. subacet., ʒi; ac. acetici diluti, ʒiv.; sp. vini rectific., ʒiv; aquæ rosæ, ad ℥viij. (*c*) R. Ammon. chlor. ℥p; ac. acet, dil., ℥fs; sp. vini rectif., ℥i; aq. ad ℥viij.

II. Allow cold water to drip from above on to a piece of lint placed over the joint. This may be readily effected by suspending over the part a wide-mouthed bottle or tin can, full of water, to which may be added ice if needed; a skein of cotton is then wetted, and one end placed in the water and the other end allowed to hang over the side of the bottle or can, when it will act as a syphon, and cause the water to pass over drop by drop (fig. 1).

Fig. 1.

III. Ice-bag applied to the part. As soon as the pain and swelling have been reduced the joint may be subjected to the same plan of treatment as has been described for the less severe cases. It only remains to consider the treatment to be pursued in those cases where, in spite of careful attention to the above details, there still remains more or less stiffness and pain. In such cases one of two methods are employed: (1) The foot is placed in a bath of hot water for about ten minutes, and then dried and subjected to manipulation or massage; (2) the adhesions are forcibly broken down if necessary under an anæsthetic, and then manipulated.

## Figures and Tables

**Fig. 1. f1:**